# Nuclear magnetic resonance-based metabolomic study of rat brain after different intensity treadmill running

**DOI:** 10.1007/s13105-025-01094-7

**Published:** 2025-05-24

**Authors:** Ni Zeng, Jie-Ting Li, Zhi-Juan Zhang, Zhi-Peng Yan, Tao Liao, Guo-Xin Ni

**Affiliations:** 1 Department of Rehabilitation Medicine, The Affiliated Hospital of Guizhou Medical University, Guiyang, China; 2 Department of Rehabilitation Medicine, Fuzhou Second Hospital, Fuzhou, China; 3 Department of Family Medicine and Primary Care, Li Ka Shing Faculty of Medicine, The University of Hong Kong, Hong Kong Special Administrative Region, Pok Fu Lam, China; 4 Department of Rehabilitation Medicine, First Affiliated Hospital of Fujian Medical University, Fuzhou, China; 5 Department of Rehabilitation Medicine, Chengdu Second People’s Hospital, Chengdu, China; 6 Department of Rehabilitation Medicine, The First Affiliated Hospital of Xiamen University, Xiamen, China

**Keywords:** Cognitive function, Running, Different running intensity, Metabolomic analysis, Hippocampus

## Abstract

Previous studies have revealed that different intensities of exercise training have an impact on cognition. However, the cognitive effects of different intensities of exercise and its underlying mechanisms are not fully understood. The aim of this paper was to investigate the effects of different intensities of treadmill exercise on cognition in rats from the perspective of metabolomic analysis. In this study, ninety-six male rats were randomly divided into four groups: control group (CON group, *n* = 24), low-intensity running group (LIR group, *n* = 24), medium-intensity running group (MIR group, *n* = 24), and high-intensity running group (HIR group, *n* = 24). After 4 weeks of treadmill running, rats in the LIR group located the platform significantly faster than those in the CON(*p* = 0.027) and HIR(*p* = 0.011) groups. After 8 weeks of treadmill running, rats in the LIR and MIR groups also found the platform more quickly than those in CON group (*p* = 0.003 and *p* = 0.015, respectively). Additionally, rats in the MIR group showed significantly increased superoxide dismutase (SOD)/catalase (CAT) in the hippocampus compared with those exposed to HIR(*p* = 0.03), LIR(*p* = 0.0008), and CON(*p* = 0.0004). Metabolomic analysis revealed that, after 8 weeks of running, 14 metabolites with similar characteristics differed between the MIR and HIR groups compared to the CON group. The LIR group showed significant alterations in 12 key metabolites compared to the CON group. The LIR, MIR, and HIR groups also demonstrated significant changes in 3, 4, and 3 metabolic pathways respectively, when compared to the CON group. In conclusion, the above results indicate that LIR can effectively decrease fumarate accumulation, thereby enhancing the TCA cycle and brain energy metabolism which in turn improved cognitive function, while MIR can modify glutathione metabolism to alleviate oxidative stress (OS), supporting cognitive function.

**Supplementary Information:**

The online version contains supplementary material available at 10.1007/s13105-025-01094-7.

## Introduction

Running is a simple intervention that helps maintain and improve cognition by inducing changes in brain neurotransmitters, neurotrophins levels, neuronal morphology, and vascularization in rodents [1]. It also enables improvement in mental states, such as depression, insomnia and stress [1–3]. In addition, previous studies have also provided robust evidence on the closed association between exercise and the hippocampus in terms of increase in cell proliferation and survival rates among rodents and hippocampal volume reversing among human beings in late adulthood [4]. Engaging in voluntary running recognized as a distinct form of exercise training has also been noted to promote neurogenesis in the hippocampus of humans, which in turn influences learning capabilities, reliant on the hippocampus memory performance, and emotional regulation [3, 5]. Exercise intensity emerges as a critical modulator of cognitive benefits [6]. Experimental studies using stressed mice (subjected to tryptophan-deficient diets and chronic unpredictable stress for 4 weeks) revealed that both continuous (20 m/min) and intermittent treadmill training (30 m/min), improved cognitive behaviors through antidepressant mechanisms [7]. High-intensity interval training (HIIT) exhibits neuroprotective properties in type 2 diabetic (T2D) rats through the modulation of hippocampal pathways that control OS, apoptosis, and protein aggregation [8–10]. The implementation of progressive treadmill training, ranging from 9 m/min to 20 m/min resulted in an increase in neural stem cell proliferation and neuronal differentiation in healthy mice [11]. Long-term moderate-intensity running maintained at 60% VO_2_max for 8 and 18 months resulted in an upregulation of upregulated brain-derived neurotrophic factor (BDNF) and serotonin levels, which subsequently enhanced cognitive function in rodents [12]. Interestingly, certain research findings suggest that prolonged periods of intense physical activity may adversely impact cognitive function. Specifically, it has been observed that seven consecutive days of exhaustive exercise result in a decline in cognitive abilities in murine models [13]. Intensive physical training and cognitive exertion can disrupt adult cognitive regulation, potentially leading to burnout syndromes [14]. The variability in findings among different studies indicates that intensity of exercise plays a crucial role in regulating cognitive function [15]. Consequently, it is crucial to delve deeper into the effects of different intensities of running on cognitive function. The intricate molecular mechanisms that drive cognitive enhancements resulting from exercise training is both complex and multifaceted [1]. The precise mechanisms through which running influence cognitive function is not yet fully understood [1–2]. A multitude of research investigations have examined the mechanisms by which physical activity improves cognitive performance [1, 16, 17]. For example, recent findings indicate that engaging in physical exercise promotes the formation of new mitochondria in skeletal muscle by activating essential regulatory molecules in a coordinated manner, such as mitogen-activated protein kinase (MAPK) and sirtuin 1 (SIRT1) [1]. In the realm of running, research indicated that engaging in voluntary running, for a duration of three months led to a decrease in astrocyte hyperplasia [16]. while simultaneously promoting an increase in synaptic density associated with astrocytes, ultimately improving cognitive function in mice afflicted with Alzheimer’s disease (AD) [16]. Additionally, research investigating the long-term impacts of various types of physical exercise on cognitive performance and OS indicators in the cerebral cortex and hippocampus [17] revealed that moderate-intensity continuous training enhanced CAT activity and facilitated improvements in recognition memory [17]. Zang et al. [18] proposed that running may improve brain function and stimulate angiogenesis through the action of endogenous nitric oxide (NO). Furthermore, various research efforts have examined the influence of physical activity on cognitive performance through a metabolic lens. Obisesan et al. [19] observed that six-months regimen of aerobic training enhanced glucose metabolism within the hippocampus of elderly individuals exhibiting mild cognitive impairment. Zhang et al. [20] illustrated that running preserved TREM2 protein levels, which were associated with improved brain glucose metabolism, and hippocampus morphological plasticity in the mice models of AD. Nevertheless, current research has predominantly concentrated on glucose metabolism, resulting in an ambiguous understanding of the contributions of alternative metabolic pathways to cognitive enhancements associated with running.

This study aimed to investigate the effects of varying intensives of running on cognitive function and the associated mechanisms involved. Considering that metabolite act as direct indicators of biochemical processes, metabolomic approaches have demonstrated efficacy in the investigation of various diseases. Here, we performed NMR-based metabolomic analysis to explore the metabolic profiles and underlying mechanisms in rats exposed to varying running intensities, with the objective of uncovering new targets for the enhancement of cognitive functions.

## Methods

### Animals

The Fujian Medical UniversityAnimal Ethics Committee gave its stamp of approval to this research. The 96 male SD rats which ranged in weight from 200 to 220 g and were 8 weeks old, were divided into four groups according to their activity level: (1) control (CON) (*n* = 24); (2) low-intensity running (LIR) (*n* = 24); (3) medium-intensity running (MIR) (*n* = 24); and (4) high-intensity running (HIR) (*n* = 24). With a total of eight participants per subgroup, we then separated the groups into three subgroups according to duration:1, 4, and 8 weeks. A 12-hour light/dark cycle and unrestricted access to food and water were provided to the rats in their controlled environment.

### Exercise training protocols

**Fig. 1:**
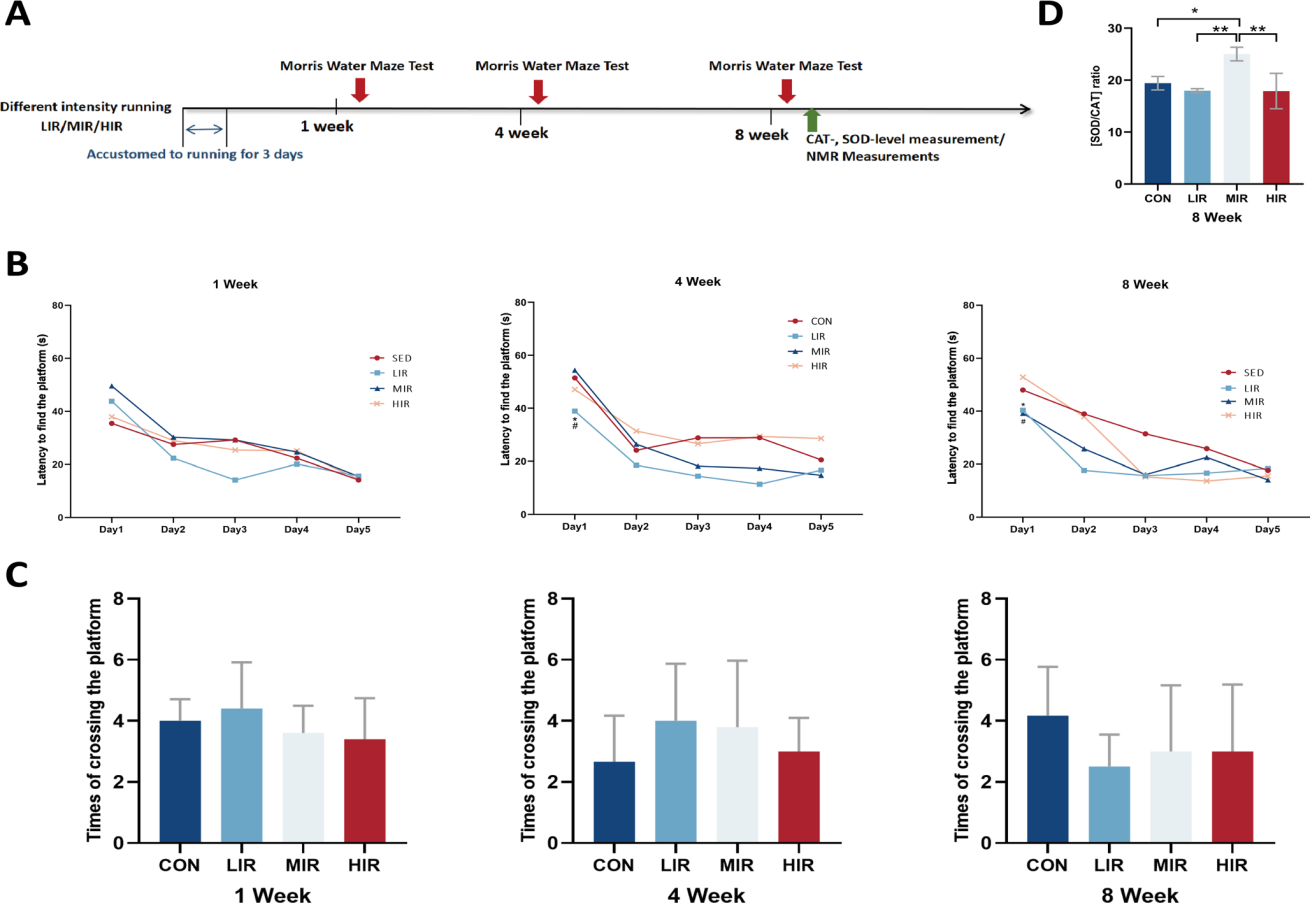
Experimental scheme (**A**). Long-term memory performance was evaluated in the Morris water maze: latency to found the platform (**B**). (*p < 0.05 compared with the control group, #*p* < 0.05 compared with the HIR group); time of crossing the platform (**C**). SOD/CAT ratio (*p < 0.05; ** *p* < 0.01) (**D**)

The rats were all subjected to 30 min of treadmill jogging at a pace of 10 m/min for one week in order to acclimate them. The intensity of the exercise intensities was adjusted in accordance with protocols that had been previously developed for treadmill use [21]. For a week, rats in the LIR, MIR, and HIR groups use a motorized treadmill once every day, Here is how the speed and inclination were set: The following speed and angle parameters are provided for the LIR MIR, and HIR: 15.2 m/min,0° inclination, MIR: 19.3 m/min,5° inclination, and HIR: 26.8 m/min, 10° inclination, and all three are valid for a duration of 60 min. For low intensity these protocols were around 40–50% for VO_2_ max, for moderate intensity, about 60–70%, and for high intensity, about 75% or more of VO_2_ max, respectively. The CON group of rats did not move around at all (Fig. 1A).

### Morris water maze (MWM) test

A digital camera and a tracking system were installed in the MWM, which was a circular tank with black inner walls and dimensions of 150 cm in circumference and 50 cm in height). The water-filled tank was 30 centimeters above ground. In the southeast corner, 2 centimeters below the water’s surface, was set up an escape platform with a diameter of 10 centimeters. In the first stage of training, rats were given 90 s to find the platform. If the rat did not reach the platform within that time, it was escorted there and given 20 s to rest before the next trial. For four days, every rat was subjected to four trials daily. On day five, we ran a test without the platform for 90 s to see how long it took to traverse to its location and how long it took to find it. The ANY-maze video tracking software (Stoelting, USA) [22] was used for data analysis.

### Brain tissue separation and collection

Rats were quickly decapitated while under the influence of isoflurane anesthesia (5% induction; 2% maintenance) then euthanized after the MWM test. Rapid brain extraction was followed by dissection. weighing, and subsequent freezing in liquid nitrogen and storage at -80 °C for the hippocampus in preparation for analysis.

### CAT- and SOD-level measurements

The levels of CAT and SOD were determined by following the instructions provided by commercially available assay kits (Beyotime Biotechnology China). After homogenizing the tissues with 100 µl of SOD Sample Preparation Solution for every 10 mg of tissue, the mixture was centrifuged at 12,000×g (4 °C, 3–5 min), and the resulting supernatant was utilized for the analysis. We followed the kit’s directions to create the reagents, which included an enzyme working solution (WST-8) and a SOD standard solution. To begin the reactions, the reaction start solution was added, stirred, and then incubated at 37 °C for 30 min. The absorbance was then measured at 450 nm. For CAT analysis, tissue homogenization was performed using the provided lysis buffer, and hydrogen peroxide standards were prepared. Absorbance at 520 nm was recorded after adding the reaction stop solution and chromogenic working solution. Enzyme activities were calculated according to the kit specifications, and the SOD/CAT ratio was derived from the normalized values.

## Metabolomics assay

### Method section

#### NMR sample preparation

Frozen hippocampus tissue was thawed on ice, and one hippocampus was removed and manually homogenized in 500 µl of ice-cold for 2 min using a plastic/Teflon homogenizer. The homogenates were then vortexed with 1875 µl of a solution consisting of 99% methanol, 98% chloroform, 36% HCl, and a ratio of 40:20:1 (v/v). Following the addition of 625 µl chloroform, the mixture was vortexed for an additional minute. Subsequently, 625 µl of water was added, and the mixture was vortexed for one more minute. The resulting solution was centrifuged at 2000 × g for 15 min, producing three distinct phases. The water/methanol layer was diluted with water and lyophilized overnight before resuspending in 2 mL of water for further metabolomic analysis. This method was adapted from previously described method [23].

#### NMR measurements

NMR measurements were conducted at 298 K using a Bruker Avance III 850 MHz NMR spectrometer that includes a BBFO cryoprobe (Bruker BioSpin, Rheinstetten, Germany).

One-dimensional (1D) ^1^H spectra were acquired using the NOESYGPPR1D pulse sequence with the following parameters: relaxation delay (RD) 4 s, short delay (t) 4 s, mixing time (τm) 10 ms, spectral width 20 ppm, acquisition time 1.88 s, and 128 transients. Chemical shifts were calibrated using TSP, set to 0 ppm.

Two-dimensional (2D) ^1^H^13^C heteronuclear single quantum coherence (HSQC) spectrum was acquired with spectral widths of 10 ppm for ^1^H and 110 ppm for ^13^C, using a 1024 × 256 data matrix and a relaxation delay of 1.5 s.

For 2D total correlation spectroscopy (TOCSY) spectra, a spectral width of 10 ppm was maintained in both dimensions with a 2048 × 256 data matrix and a relaxation delay of 1.5 s [24].

### Analysis method section

#### NMR data preprocessing

Using MestReNova (Mestrelab Research S.L., Santiago de Compostela, Spain, version 9.0), all 1D ^1^H NMR spectra adjusted for phase, baseline, and resonance. The spectral range of 5.2–4.7 ppm was omitted from the study in order to exclude the influence of residual water signals. To take any concentration fluctuations into consideration, peak integrals were standardized relative to overall peak integrals. For the purpose of statistical analysis, the 1D ^1^H spectral range (δ 0.7–4.7 and 5.0–9.0 ppm) was partitioned into bins that were 0.002 ppm wide. The relative concentration of the respective metabolites was determined by the relative integral values of independent spectral peaks. Resonances that were either singlet or non-overlapping in each spectrum were chosen for metabolite integrals calculations when pairwise comparisons between groups were performed [25].

#### Multivariate and univariate statistical analysis

The software SIMCA-*P* + 14.0 from Sartorius Stedim Data Analytics AB was used to conduct the multivariate analysis. In order to improve the signals from low-abundance metabolites, the data were scaled using Pareto scaling. To see how the groups’ internal metabolism clustered, we used Principal component analysis (PCA). For better group separation and to discover metabolite factors contributing to discrimination we used Supervised partial least squares discriminant analysis (PLS-DA) and orthogonal PLS-DA (OPLS-DA). Permutation tests (200 iterations) were used to assess the model’s resilience. Metabolites were deemed important in verified OPLS-DA models if their VIP score was greater than 1.

Metabolite concentration estimates were performed using MATLAB R2015b with normalized spectral data supplied. An analysis of variance was conducted using SPSS 19.0 developed by IBM, in the USA. Metabolites that showed *P* < 0.05 were categorized as differentially expressed, and statistical significance was determined using either student’s t-test for two groups or one-way ANOVA with Bonferroni correction for multiple groups [25, 26].

#### Metabolic pathway analysis

The analysis of metabolic pathway analysis was utilizing the MetaboAnalyst 5.0 webserver. A combination of metabolite set enrichment evaluation (*p* < 0.05) and pathway topological analysis (pathway impact value > 0.2) was used to identify metabolic pathways that were significantly altered [25, 26].

## Results

### Low and moderate running improves cognitive function

The MWM test was utilized to evaluate spatial learning and memory, which are linked to the activity of the hippocampus. Platform crossing time and latency to find the platform were not different between the running groups and the control group after 1 week of treadmill running. After four weeks, the LIR group of rats was quicker to locate the platform compared to the control (*p* = 0.027) and HIR (*p* = 0.011) group rats.

Both the LIR and MIR group rats spent significantly less time after 8 weeks of treadmill running than the control group rats (*p* = 0.003 and *p* = 0.015, respectively) (Fig. 1B). Platform crossing durations did not differ between groups at 1, 4, and 8 weeks (Fig. 1C). These findings point to the fact that after 8 weeks of low and moderate treadmill running, participants’ spatial learning and memory capacities significantly improved.

### Moderate-intensity running alleviation OS in the hippocampus

The ratio of SOD to CAT was measured to evaluate the effects of varying running intensities on OS in the hippocampus following 8 weeks of running. The SOD/CAT ratio in the hippocampus was noticeably greater in the MIR group of rats (*p* < 0.05). Based on these results, it is reasonable to assume that MIR triggers the antioxidant enzymes’ coordinated defense mechanisms (Fig. 1D).

### Metabolic profile analysis of rat hippocampus

**Fig. 2:**
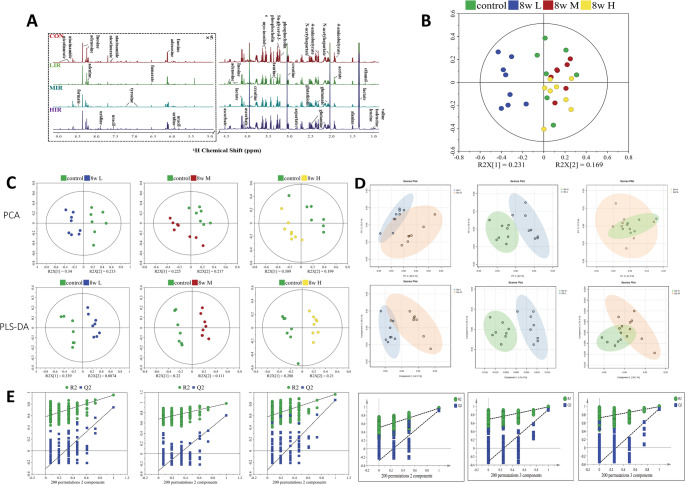
Mean NMR spectra for samples from rat hippocampus with control group (red), LIR group (green), MIR group (cyan), and HIR group (violet) (**A**). PCA score plots of ^1^H NMR data for metabolites extracted from CON, LIR, MIR, and HIR group rat hippocampus (**B**). Pair-wise PCA and PLS-DA score plots of the control group vs. LIR, MIR, and HIR group (**C**). Pair-wise PCA and PLS-DA score plots of LIR vs. MIR group, LIR vs. HIR group, and MIR vs. HIR group (**D**). Validations for pair-wise comparisons of these groups (**E**)

Figure 2 A shows representative ¹H-NMR spectra of brain from the four groups, with 2D ¹H-¹³C HSQC (Supplementary Material 2: S-Fig.1) and ¹H-¹H TOCSY (Supplementary Material 2: S-Fig.2) spectra acquired.Unsupervised PCA was conducted on the NMR data to meticulously examine the metabolic profiles of rats in both the control and running groups following an 8-week exercise regimen. The results (Fig. 2) demonstrate the various metabolic modes exhibited by the samples. Untargeted PLS-DA, accompanied by response permutation tests conducted over 200 iterations, was employed to assess the robustness of the PLS-DA models’ reliability (Fig. 2E).

The PCA score plots demonstrated clear metabolic profiles differentiating the CON group from the running groups (Fig. 2B). The score plots generated by both PCA and PLS-DA distinctly delineated the CON group from the various intensity running groups, indicating differing metabolic profiles (Fig. 2C). The LIR group demonstrated the most pronounced metabolic variations when contrasted with the CON group. Additionally, in the PCA and PLS-DA visualizations, the metabolic profiles of the LIR group exhibited some degree of differentiation from those of the MIR and HIR groups. while the MIR group showed no significant metabolic distinctions when compared to the HIR group (Fig. 2D).

### Identification of significant metabolites

**Fig. 3:**
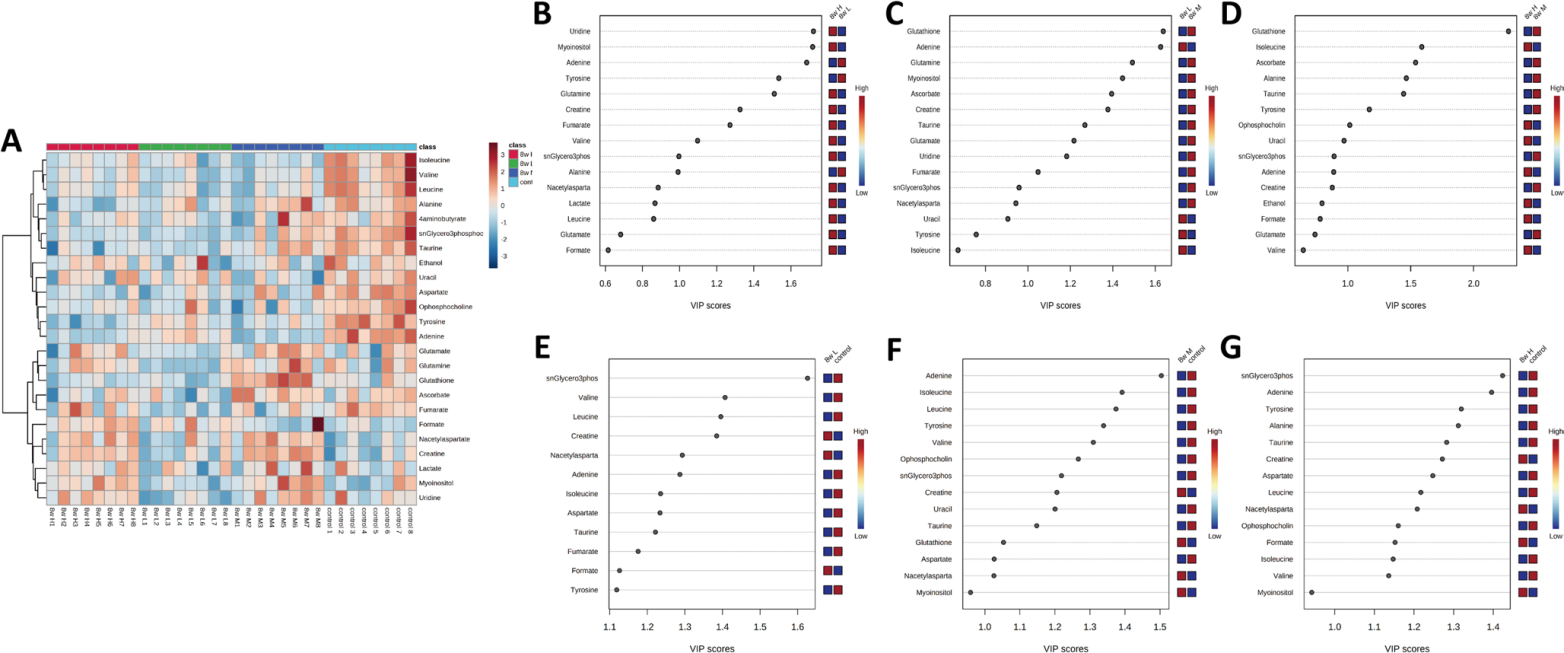
Heat map of the significantly changed metabolites between the four groups (**A**). VIP rank score of quantified metabolites of LIR vs. CON group (**B**). MIR vs. CON group (**C**). HIR vs. CON group (**D**). LIR vs. HIR group (**E**). LIR vs. MIR group (**F**). HIR vs. MIR group (**G**)

Notable variations in metabolites were detected among the running groups (Fig.3A). Table 1 (Supplementary Material 1) lists characteristic metabolites identified in rat brain samples across different running intensity groups presenting mean ± SD values and statistical significance. Through the application of OPLS-DA analysis, metabolites demonstrating notable variations were discerned, characterized by a VIP score exceeding 1.0 and a *p*-value below 0.05 as determined by two-tailed student’s t-test. Following an 8-week regimen of low-intensity running, a total of 12 metabolites exhibited values surpassing the threshold of VIP > 1.0 and *p* < 0.05. Within this dataset, 3 metabolites exhibited elevations, whereas 9 demonstrated reductions compared with the control group (Fig. 3B). The MIR group displayed 14 metabolites that significantly differed from the MIR group and control group were adenine, isoleucine, leucine, tyrosine, valine, O-phosphocholine, GPC, creatine, uracil, taurine, glutathione, aspartate, N-acetylaspartate, and myo-inositol (Fig. 3C). Furthermore, the HIR group exhibited notable changes in 14 characteristic metabolites compared with the control group (Fig. 3D).

Differences were also found in metabolites between the running groups. Fifteen different identical characteristic metabolites were found between the LIR and HIR groups (Fig. 3E). Additionally, the LIR and HIR groups exhibited 15 distinct metabolites in comparison to the MIR group (Fig. 3F and G).

### Metabolic pathway analysis results

**Fig. 4:**
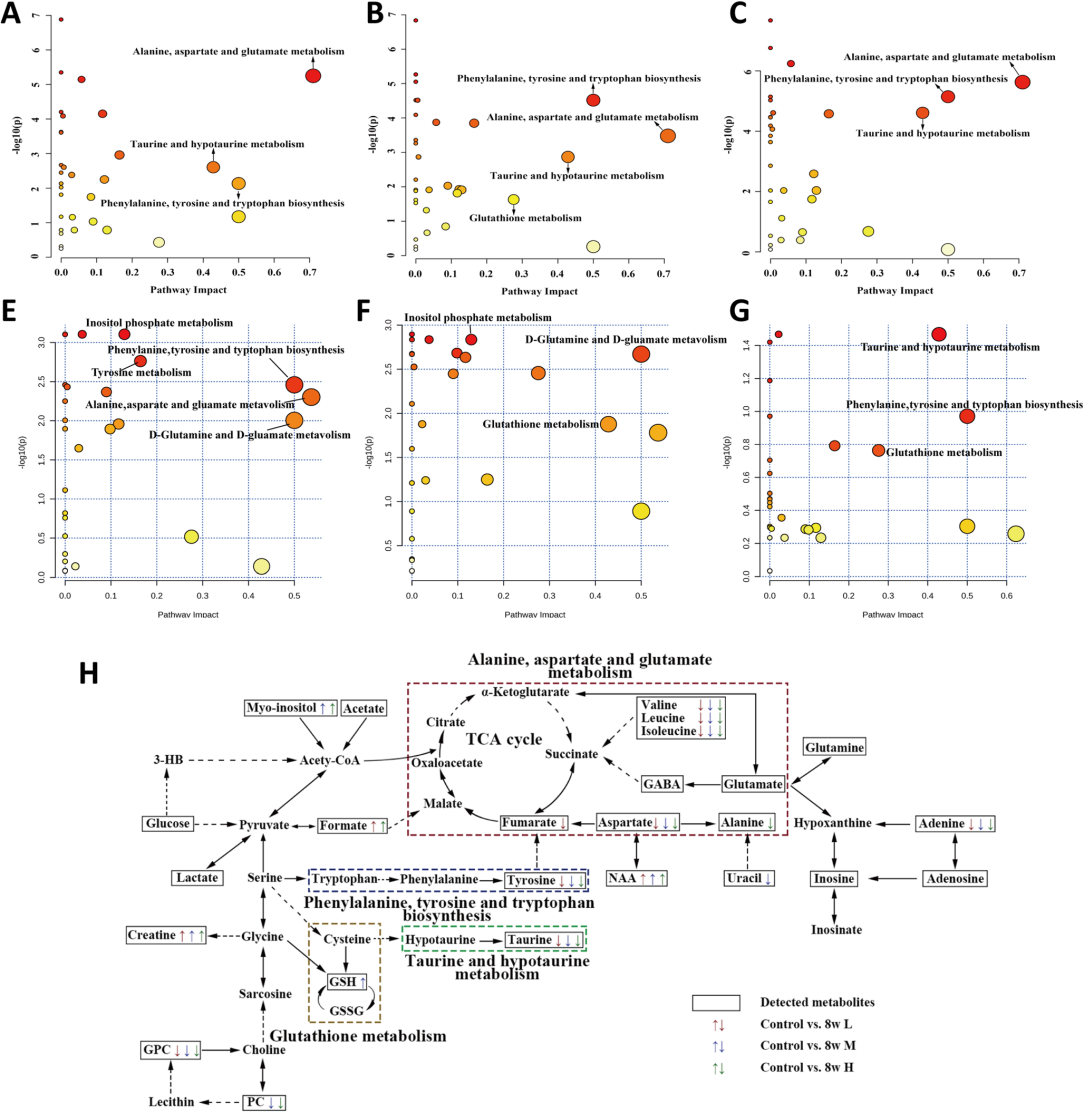
Metabolic pathway analysis (**A**). LIR vs. CON group (**B**). MIR vs. CON group (**C**). HIR vs. CON group (**D**). LIR vs. HIR group (**E**). LIR vs. MIR group (**F**). HIR vs. MIR group. A bubble represents an identified metabolic pathway, and the bubble size is proportional to the PIV, with the color denoting the statistical significance p from highest (in red) to lowest (in white). Metabolic pathways with a *p* value < 0.05 and PIV > 0.05 were identified to be significantly altered metabolic pathways. Overview of significantly altered signaling pathways and metabolic pathways (**G**). Metabolites downregulated are shown in the upward arrow, whereas those upregulated are shown in the downward arrow

Pathway impact value (PIV) > 0.2 and *p*-values < 0.05 were deemed indicative of considered significantly modified metabolic pathways. The LIR, MIR, and HIR groups exhibited notable changes in 3, 4, and 3 metabolic pathways, respectively when compared to the CON group, t (Fig. 4A, B and C). The metabolic pathways for alanine, aspartate, and glutamate metabolism; taurine and hypotaurine metabolism; and phenylalanine, tyrosine, and tryptophan biosynthesis were all shared by LIR vs. CON and HIR vs. CON, respectively.

In comparison to the LIR and HIR groups, the MIR group showed notable changes in three and two metabolic pathways, respectively (Fig. 4E, F and G). There were five noteworthy metabolic pathways that the LIR group showed in comparison to the HIR group. In order to see how the distinctive metabolites relate to the drastically changed metabolic pathways, a metabolic route analysis schematic was drawn up using data from the KEGG database (Fig. 4H).

## Discussion

Our study revealed that low- and moderate-intensity running differentially improved cognitive function in rats, accompanied by distinct metabolic alterations in the hippocampus. The significance of exercise intensity in regulating brain metabolism and oxidative stress is highlight by these findings. Below, we discuss the potential mechanisms underlying these effects, with a focus on key metabolites and the biological rationale of the training protocols.

### Low-intensity running enhances cognitive function via TCA cycle activation

Metabolomic analysis revealed reduced fumarate accumulation in the LIR group compared to controls. As a TCA cycle intermediate, fumarate accumulation is recognized as a biomarker of mitochondrial dysfunction, associated with aconitase inhibition and impaired ATP synthesis [2, 27]. The observed decrease in fumarate levels suggests enhanced TCA cycle flux in LIR rats, potentially restoring mitochondrial energy production and increasing ATP availability. This restoration is critical for cognitive function, as mitochondrial bioenergetics regulate neuroplasticity through multiple mechanisms: (1) ATP-dependent processes such as NMDA receptor activation and calcium signaling are essential for long-term potentiation (LTP) [28]; (2) mitochondrial calcium buffering facilitates organized microtubule polymerization during axonal development [2, 28]; and (3) mitochondrial impairment disrupts bioenergetic output, calcium homeostasis, and redox balance, collectively compromising cognitive performance [2, 28].

The upregulation of anaplerotic pathways (e.g., alanine, aspartate, and glutamate metabolism) in LIR rats further supports TCA cycle optimization. These pathways replenish key intermediates such as α-ketoglutarate and oxaloacetate, ensuring sustained energy production during prolonged exercise [29]. This metabolic adaptation aligns with evidence from pathological models: diabetic mice with hippocampal TCA cycle suppression exhibit spatial memory deficits [29], and Alzheimer’s patients show analogous metabolic dysregulation [30]. Collectively, these findings indicate that LIR enhances cognitive function by improving mitochondrial efficiency and energy metabolism.

### Moderate-intensity running mitigates oxidative stress through glutathione metabolism

Compared to controls, MIR rats exhibited elevated glutathione (GSH) levels and increased SOD/CAT ratios, indicative of enhanced antioxidant capacity. The enzymatic system—SOD catalyzes superoxide radical (O_2_⁻) conversion to hydrogen peroxide (H_2_O_2_), while CAT detoxifies H_2_O_2_ into water and oxygen—synergistically reduces oxidative damage to lipids, proteins, and DNA, preserving hippocampal integrity [31]. Complementing this enzymatic defense, GSH serves as the primary cellular antioxidant, directly scavenging reactive oxygen species (ROS) and regenerating oxidized molecules (e.g., vitamin C and E), thereby maintaining redox homeostasis [32]. Beyond ROS neutralization, elevated GSH modulates redox-sensitive pathways (e.g., NF-κB and MAPK), suppressing neuroinflammation and enhancing synaptic plasticity [32]. These adaptations are particularly relevant in neurodegenerative contexts: GSH depletion is a hallmark of MCI and Alzheimer’s disease [33], while exercise-induced GSH upregulation correlates with improved cognitive outcomes in both rodents and humans [31]. Thus, MIR establishes a neuroprotective milieu through dual antioxidant strategies, aligning with its observed cognitive benefits.

### High-intensity running lacks cognitive benefits: a threshold effect??

Despite inducing metabolic adaptations, HIR failed to improve cognitive performance in our study. This may reflect a threshold effect, wherein excessive ROS production during continuous HIR overwhelms antioxidant defenses, triggering neuroinflammation and mitochondrial damage [34]. Mechanistically, sustained high-intensity treadmill running upregulates hippocampal JNK/p38/ERK signaling pathways associated with neuronal inflammation, impairing spatial learning and hippocampal LTP [35]. Additionally, HIR reduces phosphorylation of cAMP-response element-binding protein (p-CREB), mature BDNF, and tropomyosin receptor kinase B (TrkB)—key mediators of synaptic plasticity and cognitive enhancement [36].

Contrastingly, HIIT improves cognition in some studies [8, 37], likely due to intermittent recovery periods that allow redox balance restoration. Our continuous HIR protocol lacked such recovery intervals, potentially exacerbating oxidative stress. The findings of this research highlight the critical significance of both the type of exercise performed and the specific dosing involved in achieving optimal neuroprotective effects.

This study has several possible limitations. Our training protocols were designed based on Bedford et al. [21]. (1979)’s standardized treadmill tests, ensuring physiological relevance. However, the lack of direct VO_2_ max measurements limits precise intensity calibration. Future studies should integrate gas analysis to validate intensity thresholds. Additionally, while metabolomics revealed pathway alterations, the absence of molecular data (e.g., BDNF, PGC-1α) restricts mechanistic depth. Moreover, we did not evaluate the changes in blood metabolites after 8 weeks of running with different intensities. On the other hand, this study only used the Morris water maze test to assess cognitive functioning, behavioral analysis methods to test spatial learning and memory should also be added.

In conclusion, low- and moderate-intensity running improve cognition through distinct mechanisms: LIR enhances energy metabolism by reactivating the TCA cycle, while MIR bolsters antioxidant defenses via glutathione metabolism. These findings emphasize the need for personalized exercise prescriptions based on metabolic and oxidative stress profiles. Future research should investigate: (1) The role of intermittent vs. continuous training in optimizing redox balance. (2) The temporal dynamics of metabolic adaptations during long-term exercise. (3) The Impact of Modulating Mitochondrial Energy Metabolism on Neurological Health. By addressing these questions, we can better harness exercise as a therapeutic strategy for cognitive decline.

## Electronic supplementary material

Below is the link to the electronic supplementary material.

Supplementary Material 1

Supplementary Material 2

## Data Availability

No datasets were generated or analysed during the current study.
