# Supplementary material for: Nuclear magnetic resonance-based metabolomic study of rat brain after different intensity treadmill running
Source: J Physiol Biochem. 2025 May 24;81(3):687–97. doi: 10.1007/s13105-025-01094-7 (PMC12373686; doi:10.1007/s13105-025-01094-7)
Supplement: Supplementary file 1 — Supplementary Material 1 [file 13105_2025_1094_MOESM1_ESM.pdf]

Table 1. Characteristic metabolites identified in different running group of rat brain

Metabolite	Mean ± SD				Student's test					
	Control	8w L	8w M	8w H	Control vs. 8w L	Control vs. 8w M	Control vs. 8w H	8w L vs. 8w M	8w L vs. 8w H	8w M vs. 8w H
Valine	0.086±0.011	0.063±0.007	0.061±0.006	0.067±0.006	***	***	**	NS	NS	NS
Isoleucine	0.038±0.006	0.027±0.006	0.022±0.003	0.028±0.003	**	***	**	NS	NS	**
Leucine	0.339±0.029	0.262±0.027	0.246±0.023	0.271±0.019	***	***	***	NS	NS	NS
Ethanol	0.039±0.007	0.036±0.012	0.031±0.005	0.036±0.005	NS	*	NS	NS	NS	NS
Lactate	2.603±0.300	2.620±0.360	2.659±0.415	2.744±0.210	NS	NS	NS	NS	NS	NS
Alanine	0.752±0.032	0.730±0.053	0.693±0.054	0.630±0.039	NS	*	***	NS	*	*
4-aminobutyrate	2.043±0.246	1.807±0.121	1.781±0.430	1.765±0.174	*	NS	*	NS	NS	NS
N-acetylaspartate	4.872±0.310	5.383±0.413	5.376±0.386	5.462±0.317	*	*	**	NS	NS	NS
Glutamate	0.430±0.037	0.446±0.027	0.445±0.027	0.438±0.034	NS	NS	NS	NS	NS	NS
Glutamine	0.229±0.072	0.168±0.036	0.246±0.062	0.234±0.045	NS	NS	NS	*	*	NS
Glutathione	0.054±0.008	0.052±0.007	0.066±0.008	0.048±0.004	NS	**	NS	**	NS	****
Aspartate	0.416±0.030	0.370±0.024	0.353±0.049	0.346±0.029	**	**	***	NS	NS	NS
Creatine	5.293±0.269	5.700±0.276	5.804±0.242	5.828±0.203	*	**	**	NS	NS	NS
O-phosphocholine	1.157±0.076	1.029±0.155	0.866±0.151	0.995±0.071	NS	***	**	NS	NS	NS
GPC	1.482±0.103	1.064±0.060	1.153±0.186	1.079±0.057	***	**	***	NS	NS	NS
Taurine	3.074±0.138	2.762±0.176	2.780±0.149	2.551±0.227	**	**	***	NS	NS	NS
Myo-inositol	2.790±0.219	2.889±0.111	3.068±0.172	3.117±0.195	NS	*	**	NS	*	NS
Ascorbate	0.436±0.032	0.423±0.066	0.452±0.056	0.413±0.029	NS	NS	NS	NS	NS	NS
Uracil	0.014±0.001	0.013±0.002	0.010±0.002	0.012±0.003	NS	**	NS	*	NS	NS
Uridine	0.014±0.002	0.013±0.002	0.015±0.001	0.015±0.001	NS	NS	NS	NS	NS	NS
Fumarate	0.034±0.004	0.027±0.004	0.029±0.005	0.031±0.006	**	*	NS	NS	NS	NS
Tyrosine	0.052±0.006	0.043±0.005	0.035±0.0045	0.031±0.006	**	***	***	*	**	NS
Adenine	0.085±0.009	0.071±0.005	0.047±0.005	0.053±0.006	**	***	***	****	***	NS
Formate	0.007±0.001	0.008±0.001	0.007±0.003	0.009±0.001	*	NS	**	NS	NS	NS

Data are presented as the means ± SD, Statistical significance: $*p<0.05$， $**p<0.01$， $***p<0.001$; GPC, sn-Glycero-3- phosphocholine.
