# Supplementary material for: Nuclear magnetic resonance-based metabolomic study of rat brain after different intensity treadmill running
Source: J Physiol Biochem. 2025 May 24;81(3):687–97. doi: 10.1007/s13105-025-01094-7 (PMC12373686; doi:10.1007/s13105-025-01094-7)
Supplement: Supplementary file 2 — Supplementary Material 2 [file 13105_2025_1094_MOESM2_ESM.pdf]

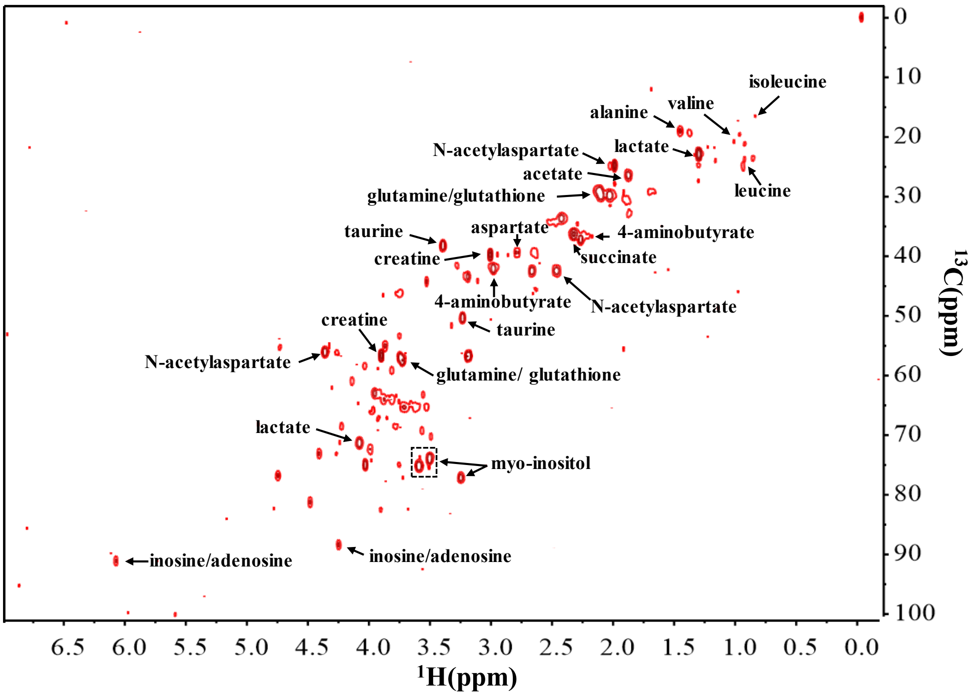

S-Fig.1 $^{1}H^{13}C$ heteronuclear single quantum coherence (HSQC) spectrum

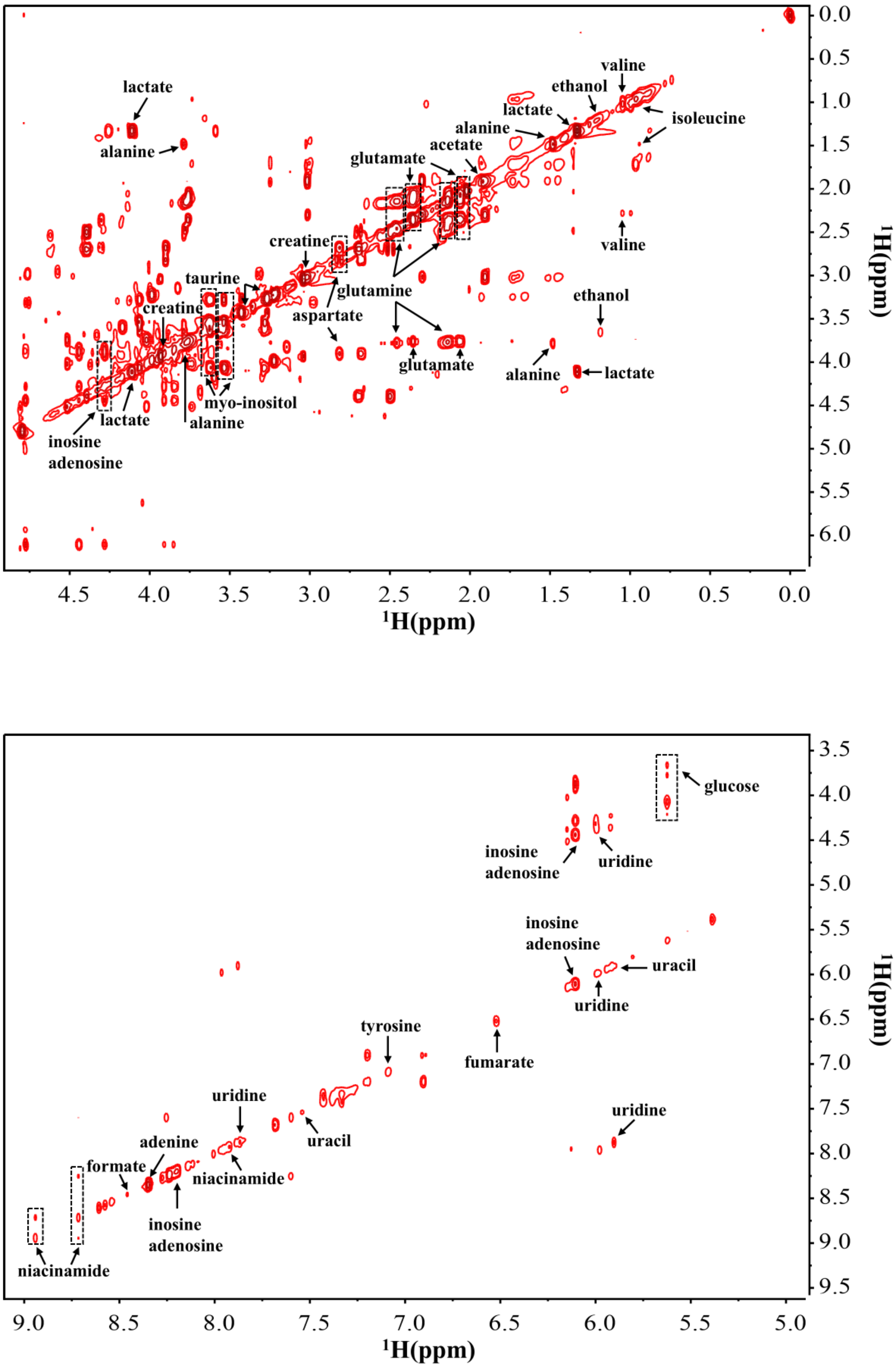

S-Fig.2 2D ^{1}H-^{1}H total correlation spectroscopy (TOCSY) spectrum
